# Hypoxia Alleviating PdTe Nanoenzymes for Thermoradiotherapy

**DOI:** 10.3389/fbioe.2021.815185

**Published:** 2022-03-11

**Authors:** Yang Li, Xinquan Gu, Fan Yu

**Affiliations:** ^1^ Department of Urology, China-Japan Union Hospital of Jilin University, Changchun, China; ^2^ Department of Gartroenterology and Hepatology, China-Japan Union Hospital of Jilin University, Changchun, China

**Keywords:** nanoenzyme, radiotherapy, hypoxia, thermoradiotherapy, tumor microenvironment

## Abstract

Hypoxia in the tumor microenvironment induces radioresistance in cancer cells, which reduces the treatment efficiency of radiotherapy. Therefore, it is critical to produce sufficient oxygen to alleviate hypoxia to enhance the effect of ionizing radiation. Here, we constructed nanorod-shaped PdTe nanoenzymes to overcome hypoxia and promote the effects of thermoradiotherapy. Both palladium and tellurium are high-Z elements, which interacted with X-rays to generate more DNA radicals in the tumor regions. Moreover, PdTe nanoenzyme could catalyze the conversion of intratumoral overexpressed H_2_O_2_ to oxygen, alleviating hypoxia in the tumor regions. Photothermal therapy mediated by PdTe nanoenzymes not only ablated tumors but also accelerated the blood flow, in turn, modulating hypoxia. With good biocompatibility, PdTe nanoenzyme exhibited remarkable oxygen generation ability both *in vitro* and *in vivo*, indicating potential ability for radiosensitization. Further investigation using MBT-2 cells and MBT-2 tumor-bearing mice demonstrated that PdTe nanoenzyme could effectively enhance the treatment efficiency of radiotherapy. Thus, our work presented a novel nanoenzyme to overcome hypoxia in tumors for effective thermoradiotherapy.

## Introduction

The clinical treatment of cancer involves radiotherapy as a conventional method, which is conducted either alone or in combination with other therapeutic methods (Laprise-Pelletier et al., 2018; Zhu et al., 2020b; Sasieni and Sawyer 2021; Sung et al., 2021; Zheng et al., 2021). Radiotherapy induces the apoptosis of cancerous cells by inducing a direct or indirect damage to their DNA using α, β, γ rays, X rays, and other particle beams. However, the treatment efficiency of radiotherapy might be influenced by various factors, including the researcher’s experience, daily changes in the patient’s position when receiving treatment, tumor proliferation, and the motion of uncontrollable breath (Raaymakers et al., 2017; Gao et al., 2020; Sasieni and Sawyer 2021). Hereby, we tried to improve the accuracy of this treatment methodology. Adaptive radiotherapy, as a type of image-guided therapy, is a novel strategy that requires frequent measurements of the location of tumors and updated plans to better monitor the changes in tumor size (McPartlin et al., 2016; Nabavizadeh et al., 2016; Castelli et al., 2018). However, this strategy might expose the patient to excessive doses induced by frequent computed tomography scans and heavy work for planners (Nabavizadeh et al., 2016). Moreover, considering the possibility of tumor infiltration, adjacent normal tissues can inevitably be included in the tumor-targeting area during the radiotherapy, leading to severe side effects as well as causing pain to patients (Bruheim et al., 2010; Zhu et al., 2020b; Du et al., 2021; Zheng et al., 2021). Hence, it remains an issue to improve the efficacy of radiotherapy on tumors while avoiding side effects on normal tissues and organs.

Cancerous cells possess the characteristic of uncontrolled proliferation, which leads the tumor to “outgrow” its nutrition and oxygen supply (Finger and Giaccia, 2010; Yoon et al., 2018; Chen et al., 2019). Thus, tumors reside in a special tumor microenvironment—mild acidity, hypoxia, and overexpressed H_2_O_2_ (Lu et al., 2018). Hypoxia in tumors causes radioresistance, impairing the treatment efficiency of radiotherapy (Song et al., 2017; Laprise-Pelletier et al., 2018). Thus, radiotherapy induces cancer cell apoptosis by DNA double-strand breaks. However, the DNA radicals can be repaired by the cancer cell itself. Thus, oxygen is necessary for “fixing” these DNA radicals to prevent this repair, to ensure that oxygen can interact with DNA radicals to convert these damages to permanent ones (Yi et al., 2016; Zheng et al., 2021). Thus, alleviating hypoxia could efficiently improve the effect of radiotherapy (Xia et al., 2020; Zhu et al., 2020a).

Recent studies have been interested in nanoplatforms designed for radiosensitization. Nanoparticles with high Z elements, such as gold (Sun et al., 2020), bismuth (Cheng et al., 2017; Deng et al., 2018), and gadolinium have been shown to emit high-energy radiations on tumor cells. Despite those nano-radiosensitizers, nanoparticles that modulate the tumor microenvironment provide another problem-solving strategy. Some nanoplatforms based on hemoglobin or perfluorocarbon (PFC) possess the ability to deliver oxygen to tumors to modulate hypoxia (Gao et al., 2017; Xia et al., 2020). Previous studies have reported that Bi_2_Se_3_ hollow nanoparticles-loaded PFC nanoplatforms can cause *in situ* release of oxygen in tumors (Cheng et al., 2017). Considering that an excessive amount of endogenic H_2_O_2_ exists in tumorous cells, nanoenzymes that can catalyze this overexpressed H_2_O_2_ to oxygen to alleviate hypoxia can be used to enhance radiotherapy. MnO_2_ can convert H_2_O_2_ to oxygen, and thus, nanoplatforms, such as hollow MnO_2_ can be used to modulate the tumor microenvironment (Prasad et al., 2014; Yi et al., 2016; Zhu et al., 2016; Lyu et al., 2020b). Moreover, photothermal therapy, mediated by a photothermal agent, can not only ablate tumors to a certain extent but also improve the oxygen status in tumors by accelerating intratumoral blood flow. Hence, nanoparticles, such as bismuth dots and FePd-Cys nanodots-mediated combination therapy of photothermal therapy and radiotherapy has been shown to exhibit remarkable potential for tumor suppression (Deng et al., 2018; Lyu et al., 2020a). However, due to the ultra-small structure, these nanoparticles are cleared out of the body rapidly before conducting radiotherapy, which limits their application to radiosensitization (Liu et al., 2015; Guosheng Song et al., 2016; Lu et al., 2018).

**SCHEME 1:**
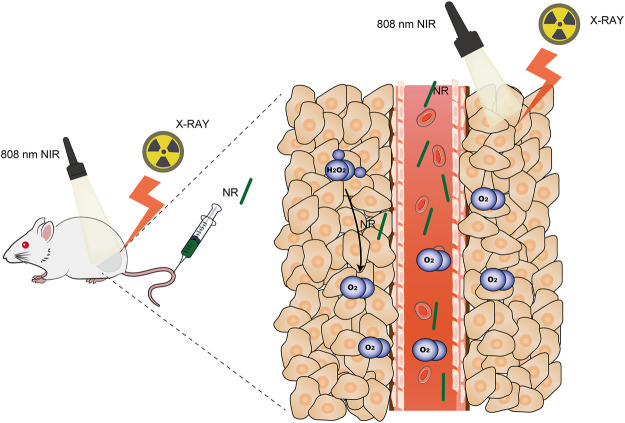
Schematic Illustration of thermoradiotherapy of NR.

Here, we synthesized a nanorod consisting of palladium and tellurium (NR) for thermoradiotherapy as shown in Scheme 1. NR acted as a photothermal agent to mediate photothermal therapy to accelerate blood flow to modify hypoxia. Since both Pd and Te are high Z elements that attribute to the photoelectric effect (Lyu et al., 2020a; Huang et al., 2020), this novel NR exhibited remarkable radiosensitization ability both *in vitro* and *in vivo*. Moreover, this nanorod could catalyze the conversion of intracellular overexpressed H_2_O_2_ in tumor regions to O_2_, which alleviated hypoxia and hereby enhanced the effect of radiotherapy. Further investigation on the therapeutic effect of PdTe nanorods conducted on MBT-2 tumor-bearing mice demonstrated the superior potential of PdTe nanorods on enhancing the effect of chemoradiotherapy.

## Experiment Section

### Synthesize of PdTe NR

Initially, 0.08 g precursor sodium tellurite (Aladdin) and 0.1 g sodium tetrachloropalladate (Aladdin) were dissolved in 100 ml absolute ethanol under sonication. Next, 1.5 ml of oleic acid and 1.5 ml of oleylamine were added in this solution under stirring. 0.6 g of NaBH_4_ was dissolved in 50 ml ethanol solution. Then the NaBH_4_ solution was added drop by drop. Then the mixture was stirred for 3 h at room temperature. After reaction, the mixture was centrifuged at 10,000 rpm for 10 min. Then the supernatant was removed and the precipitation was washed with ethanol twice. The black product was next dried in vacuum oven for 12 h to obtain PdTe NR.

### Materials Characterization

The morphology of NPs was observed by transmission electron microscopy (TEM; Tecnai G2 F20 S-Twin, FEI, United States) at 100 keV acceleration voltage. The surface chemical elements were analyzed by XPS (ESCA-Lab250XI, Thermo Fisher Ltd., United States). The optical absorbance of NPs was measured effectively by UV-Vis NIR spectrophotometer (CARY5000, Varian Ltd., United States). The phase structures were acquired using X-ray diffraction (XRD; Bruker D8 Advance, Germany) with CuKα radiation ( λ=0.15406nm ).

### *In vitro* Photothermal Experiment

PdTe NR were dispersed in 1 ml of PBS solution at a concentration of 100 μg/ml, then 1 ml of the prepared solution was irradiated with a 1064 nm near-infrared laser (MDL-N-1064, Changchun New Industries Optoelectronics Technology, China) with an energy power of 1 W/cm^2^, and the temperature changes were monitored and recorded in real-time with an IR camera thermographic system (HBT-2A, Hao Bo Technology, China). Then the recorded data was analyzed using the GraphPad software to draw a photothermal curve.

### *In vivo* Photothermal Experiment

The tumor model was established by subcutaneously injecting 100 μL (1 × 10^7^ cells/mL) of MBT-2 cell suspension into the right hind leg of 4 to 5-week-old BALB/c female mice (Vital River Company, Beijing, China). When the tumor size reached about 100 mm^3^, the mice were divided into two groups (5 mice/group). The mice in the control group and experimental group were injected through tail vein with 50 μL of PBS and 50 μL NPs (100 μg/ml), respectively. After 24 h, a 1064 nm laser with an energy power of 1 W/cm^2^ was used to illuminate the tumor sites of both groups of mice for 5 min. At the same time, an infrared camera was used to record the temperature changes in the tumor sites in real-time, and infrared thermal images were recorded. All animal experiments were approved by the Institution Animal Ethics Committee of Jilin University (license No. pzpx20210720029).

### Cell Experiment

The MBT-2 cells (the Cell Bank of the Chinese Academy of Sciences) were incubated in RPMI-1640 medium containing 10% FBS in a humidified atmosphere at 37°C with 5% CO_2_.

### Cytotoxicity Assay (CCK-8)

CCK-8 assay kit was used to evaluate the cell viability of each group. Cells (5 × 10^3^ cells/well) were seeded into 96-well plates and were divided into the following six groups (5 wells/group): group 1: Control; group 2: NR; group 3: RT; group 4: NR + NIR; group 5: NR + RT; group 6: NR + NIR + RT. After culturing for 24 h, the cells were treated according to the requirements of each group and incubated again for 24 h. Next, we added 10 μL of CCK-8 reagent to each well. After incubation for 2 h, the absorbance at 450 nm was measured using a microplate analyzer (Rayto-6000 system, Rayto, China).

### *In vitro* DNA Damage Test

DNA damage in each group of cells was labeled using γ-H_2_AX antibodies under hypoxia status. We seeded cells on coverslips and divided into five groups: group 1: Control; group 2: NR; group 3: RT; group 4: NR + NIR; group 5: NR + RT; group 6: NR + NIR + RT. Then concentration of NR was 100 μg/ml. After treatment, the old culture medium was discarded, fixed with 4% paraformaldehyde at 4°C for 15 min, and washed thrice with PBS. Triton-X-100 (0.2%) was used for film breaking at 4°C for 15 min, then kept at room temperature for 2 h, and washed thrice with PBS. The cells were stained with DAPI and secondary anti-γ H_2_AX antibodies (5% FBS and 1% Tri-X-100) and incubated in the dark. After washing with PBS, the cells were sealed with 90% glycerin and covered glass with the cells facing down. The immunofluorescence analysis of the cells was done using a fluorescence microscope (IX81, Olympus, Japan).

### *In vivo* Photoacoustic Imaging

Five tumor-bearing mice were depilated at their tumor sites to evaluate the PA imaging performance of NPs *in vivo*, and the tumor sites of mice were scanned using the Vevo® LAZR system (Fujifilm, Visualsonics Inc. Canada) to obtain the PA images. Then the mice were injected with 50 μL of NR (100 μg/ml), and the tumor sites were scanned by the same instrument at 0 and 24 h, respectively to obtain the PA images.

### *In vivo* Hypoxia Evaluation

When the tumor size reached 200 mm^3^, the mice were randomly divided into five groups (*n* = 5): group 1: Control; group 2: NR; group 3: RT; group 4: NR + NIR; group 5: NR + RT; group 6: NR + NIR + RT. For group 2, 4, 5 and 6, the mice were injected with 50 μL of NR (100 μg/ml) once. The dosage of radiation was 6 Gy. After treatment, the mice were sacrificed, and the tumors were effectively stained with HIF-1α. The subsequent processing of the tumor components of HIF-1α was achieved using the MATLAB software.

### *In vivo* Antitumor Efficacy

When the tumor size approached 100 mm^3^, 25 mice were randomly divided into five groups (*n* = 5) to receive various treatments: group 1: Control; group 2: NR; group 3: RT; group 4: NR + NIR; group 5: NR + RT; group 6: NR + NIR + RT. For group 2, 4, 5, and 6, the mice were injected with 50 μL of NR (100 μg/ml). The dosage of radiation was 6 Gy. The length and width of the tumors were measured with calipers every 3 days to record the changes in their volume and body weight. After 18 days of treatment, all mice were sacrificed, their major organs (heart, liver, spleen, lung, kidney) and tumors were removed, fixed with 4% neutral buffer formalin, and routinely processed into paraffin sections, which were stained by DAFH-DA and examined by light microscopy (BX51, Olympus, Japan).

### Biochemical Blood Analysis

When the tumor volume reached 80–100 mm^3^, the tumor-bearing mice were intravenously injected with 50 μL of NPs (100 μg/ml), and then blood samples were collected from the mouse eye socket at 0.5, 2, 4, 8, 12, and 24 h, respectively.

## Results and Discussion

**SCHEME 2:**
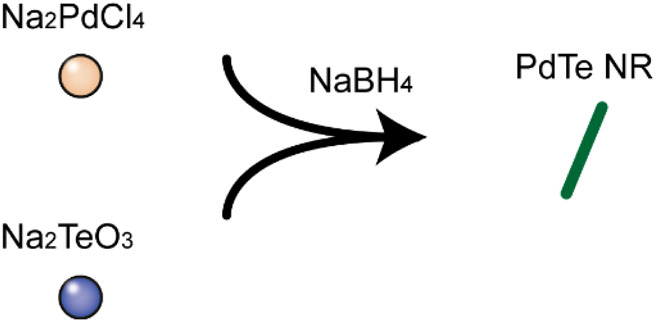
Process of PdTe NR synthesize.

**FIGURE 1:**
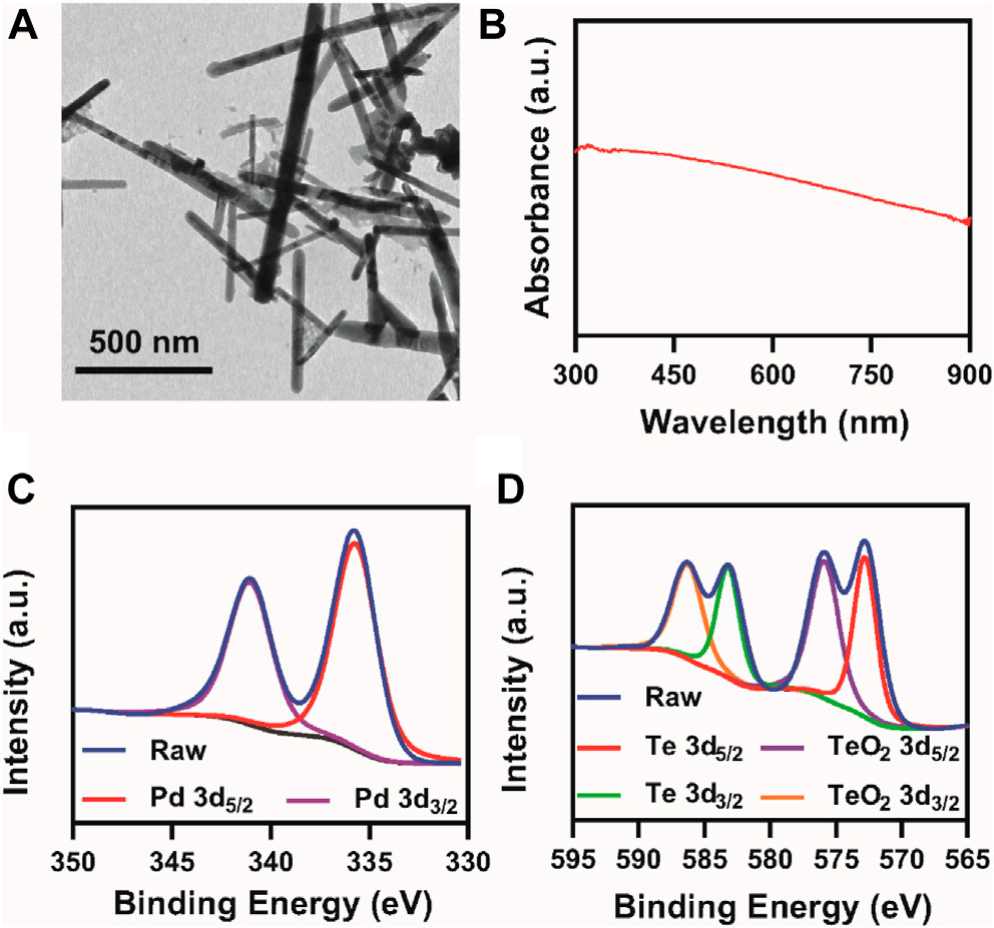
Characterization of NR. **(A)** TEM images; **(B)** UV-vis spectrum of PdTe NRs at 100 μg/ml; XPS spectra of **(C)** palladium and **(D)** tellurium.

PdTe NR was synthesized via the one-pot method as shown in Scheme 2. First, precursor sodium tellurite (0.08 g) and sodium tetrachloropalladate (0.1 g) were dissolved in absolute ethanol (100 ml), followed by the addition of 1.5 ml of oleic acid and 1.5 ml of oleylamine with stirring. After the solution became clear, 0.6 g of NaBH_4_ ethanol solution (50 ml of ethanol) was added and allowed to react for 3 h at room temperature, resulting in the formation of PdTe NR. PdTe NR was rod-shaped, and 632 ± 172 nm in length and 27 ± 4 nm in width (Figure 1A). The UV-Vis spectrum demonstrated a broad absorbance band from 300 to 900 nm, without any obvious sharp peaks (Figure 1B **)**. X-ray photoelectron spectroscopy (XPS) of NR in Supplementary Figure S1 confirmed Te 3d and Pd sd orbits. Further verification of Pd and Te orbits were conducted to further confirm the composition of NR (Figures 1C,D **)**. We observed that Pd was present in NR in the form of a simple substance; however, Te was present in both simple substance and TeO_2_. The result of X-ray diffraction (XRD) pattern, consistent with the result of XPS, in Supplementary Figure S2 suggested that NR was consisted of Pd (JSPDS file 87–0637), TeO_2_ (JSPDS file 46–1211) and Te (JSPDS file 79–0736). Moreover, the zeta potential of NR exhibited no obvious difference after 30 days observation (Supplementary Figure S3).

**FIGURE 2:**
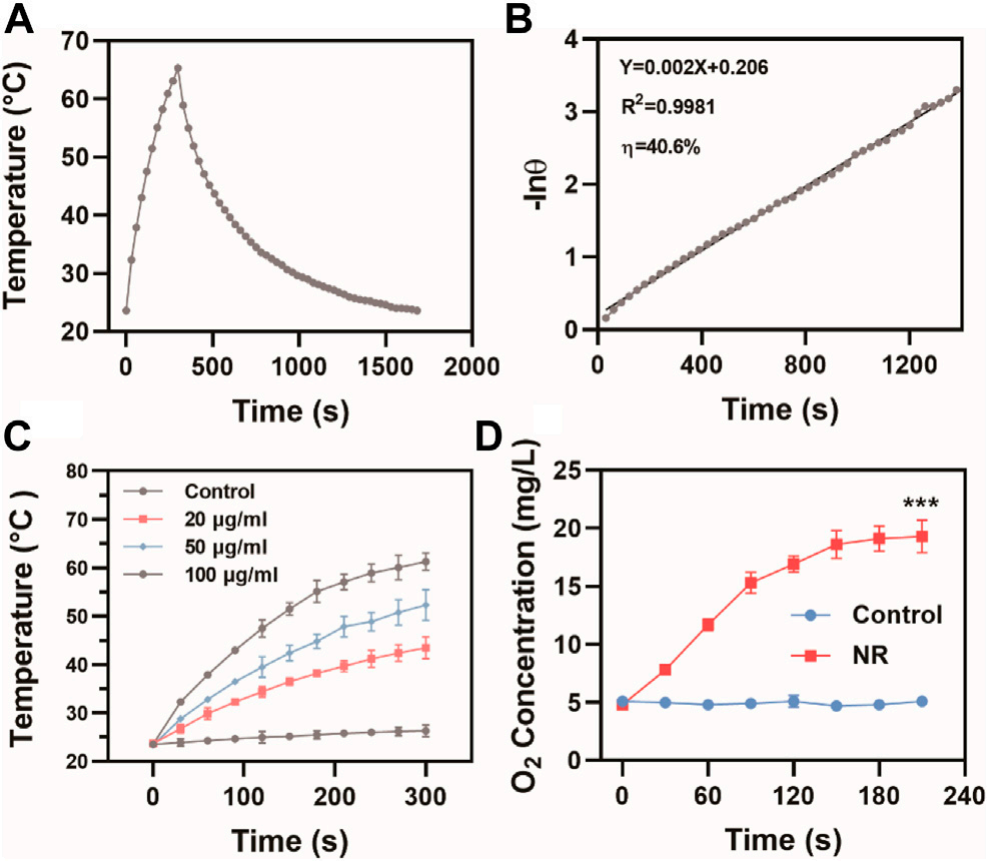
Photothermal properties of NR. **(A)** The photothermal effects and **(B)** Linear regression of the cooling profile of NR aqueous dispersions (100 μg/ml) at 808 nm laser irradiation at 1 W/cm^2^; **(C)** Temperature changes of NR solution at various concentrations at 808 nm laser irradiation at 1 W/cm^2^; **(D)** The oxygen content in H_2_O_2_ (30 mM) treated with H_2_O or NR (100 μg/ml) (*n* = 3). The data are represented as mean ± SD, ***p < 0.001.

Since both Pd and Te elements exhibited great potential in photothermal therapy, the possibility of NR as photothermal agents were assessed. At 808 nm laser irradiation at 1 W/cm^2^, the temperature of NR solution at a concentration of 100 μg/ml rose from 23.6°C to 65.3°C (Figure 2A), which met the demand for photothermal therapy. The photothermal conversion efficiency (η) of NR was calculated to be 40.6% (Figure 2B). Meanwhile, NR exhibited photothermal stability and remained photothermal conversion ability after five loops of heating and cooling cycle as shown in Supplementary Figure S4. Further investigation on the temperature rose of the various concentration in Figure 2C showed that the degree of temperature rise depended on the concentration of NR. The higher the concentration of NR was, the higher the solution temperature could reach. Moreover, due to the rapid proliferation of cancerous cells, the tumor microenvironment was hypoxic, which lead to radioresistance. Hereby, we also evaluated the potential of NR as a nanoenzyme for H_2_O_2_, which was overexpressed in the tumor site. After adding NR solution to H_2_O_2_, the oxygen content level rose dramatically from 4.8 ± 0.1 mg/L to 19.3 ± 1.4 mg/L, indicating the catalyze ability of NR on transforming H_2_O_2_ to O_2_ (Figure 2D **)**.

**FIGURE 3:**
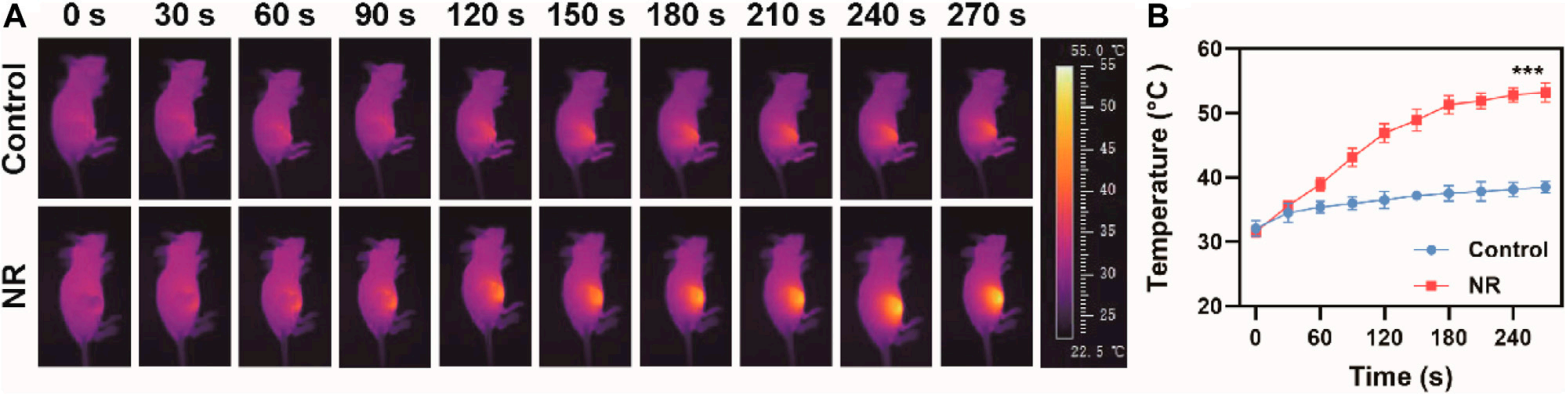
Photothermal effect *in vivo*. **(A)** Thermal images of mice after administration of PBS or NR (100 μg/ml) and **(B)** Corresponding temperature change under 808 nm irradiation (1.0 W/cm^2^). The data are represented as mean ± SD, ***p < 0.001.

Following the outstanding photothermal effect results and high photothermal conversion efficiency, we further investigated the feasibility of NR as a photothermal agent *in vivo*. Before the *in vivo* photothermal therapy experiment, biocompatibility of NR *in vivo* was examined. No obvious injuries in main organs were observed post injection of NR (Supplementary Figure S6). As shown in Supplementary Figure S5, hemolysis rate of NR is 13.4 ± 5.1%. Moreover, hematological index of mice after administration of NR was assessed in Supplementary Figure S7, suggesting no obvious toxicity of NR to mice. Next, the blood circulation and biodistribution post intravenous injection of NR was investigated. Supplementary Figure S8 shows the pharmacokinetic behavior of NR until 24 h post-injection, which was fit using a two-compartment pharmacokinetic model. The half-circulation time of NR was calculated to be 1.70 h. Then 24 h post-injection, the mice were sacrificed and the biodistribution of NR in main organs and tumors was measured using an inductively coupled plasma atomic emission spectrometer (ICP-AES). NR accumulated at a high level in the liver and a relatively high level in the spleen, as well as tumor attributed to passive targeting ability via enhanced permeability and retention effect (Supplementary Figure S9). Based on these results, we investigated *in vivo* photothermal conversion effect on MBT-2 tumor-bearing mice. After 24 h of the intravenous injection of PBS or NR via tail vein, an 808 nm laser was used to irradiate the tumor region while a thermal imaging system was used for imaging. Compared with the group in which the mice were administrated with PBS, tumor regions of the mice injected with NR exhibited remarkable temperature rise from 31.6°C to 53.2°C (Figures 3A,B).

**FIGURE 4:**
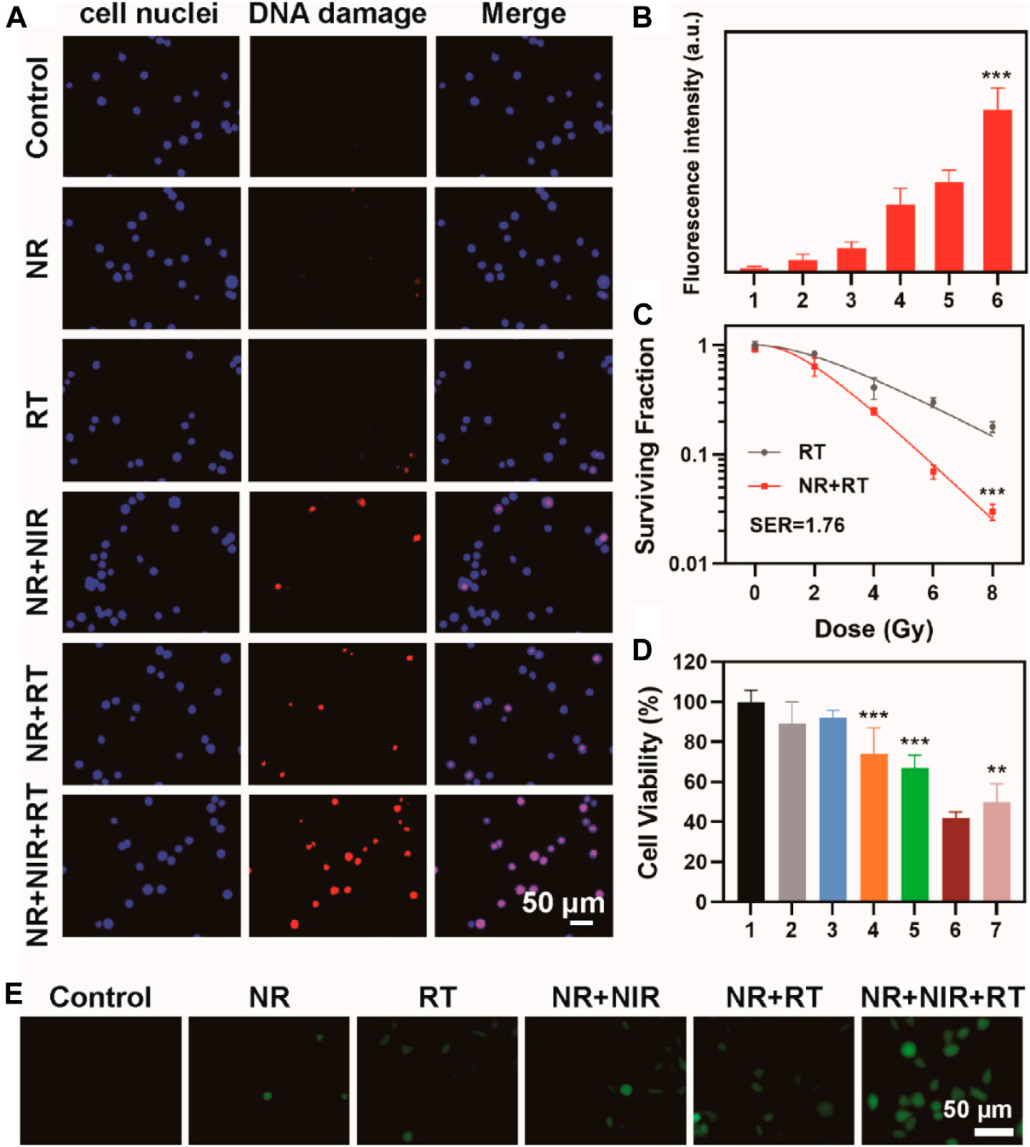
**(A)** Confocal fluorescence image of γ-H_2_AX stained MBT-2 cells after various treatments and **(B)** corresponding fluorescence analysis; **(C)** Curve fitting of colony formation assay (group 1: Control; group 2: NR; group 3: RT; group 4: NR + NIR; group 5: NR + RT; group 6: NR + NIR + RT); **(D)** Cell viability after various treatments (group 1: Control; group 2: NR; group 3: RT; group 4: NR + NIR; group 5: NR + RT; group 6: NR + NIR + RT; group 7: projective additive). **(E)** ROS detection of MBT-2 cells after various treatments. The data are represented as mean ± SD, **p < 0.01; ***p < 0.001.

Biocompatibility should be considered before the use of NR for thermoradiotherapy. After incubation with NR for 24 h at a various concentration range from 12.5 to 200 μg/ml, CCK eight assay was done to assess the cell viability of MBT-2 cells. We found that the cell viability was >80% even at a concentration of 100 μg/ml, indicating no distinct cytotoxicity of NR to MBT-2 cells (Supplementary Figure S10). Thus, further investigation on DNA damage of cells was conducted. Radiotherapy is known to induce cell apoptosis by direct or indirect DNA double-strand breaks, and thus, γ-H_2_AX staining was used to detect DNA damage. Minor DNA damage was found in the cell treated with RT; however, a relatively high level of DNA damage was observed in the group treated with NR + RT or NR + NIR. Interestingly, CLSM images of cells treated with NR + NIR + RT exhibited the highest level of red fluorescence among all the groups (Figure 4A). The fluorescence intensity of the group treated with NR + NIR + RT was 6.75 times that in groups treated only with RT, which demonstrated a remarkable cell-killing effect in DNA level (Figure 4B). As the “golden standard” for radiosensitization evaluation, cell colony formation assay was conducted, and a multi-target with single hit model was used for fitting those data. From the parameters that resulted from fitting, the sensitizing enhancement ratio (SER) of NR was calculated to be 1.76, confirming the potential of NR as a radiosensitizer (Figure 4C). Additionally, cell viability after various treatments were assessed using CCK eight assay to further confirm the effect of thermoradiotherapy mediated by NR. Cells in group 1, group 2, and group 3 exhibited slight damage (Figure 4D); however, the cell viability in group 4 and group 5 decreased to a certain extent. Compared to group 4 and group 5, the cell viability dropped sharply to roughly 42.0%. Here the group of projective additive value was the product of cell viability in group 4 and group 5, which represented the additive effect of radiotherapy and photothermal therapy of NR. It should be noted that group 6, in which cells were treated with NR + NIR + RT, exhibited more effective cell death, indicating the synergistic effect of NR mediated radiotherapy and photothermal therapy. To further detect the reactive oxygen species (ROS) produced in cells, MBT-2 cells were subjected to various treatments. As shown in Figure 4E, fluorescence intensity of NR + NIR + RT groups was the highest among all groups, indicating largest amount of ROS generation of NR mediated thermoradiotherapy.

**FIGURE 5:**
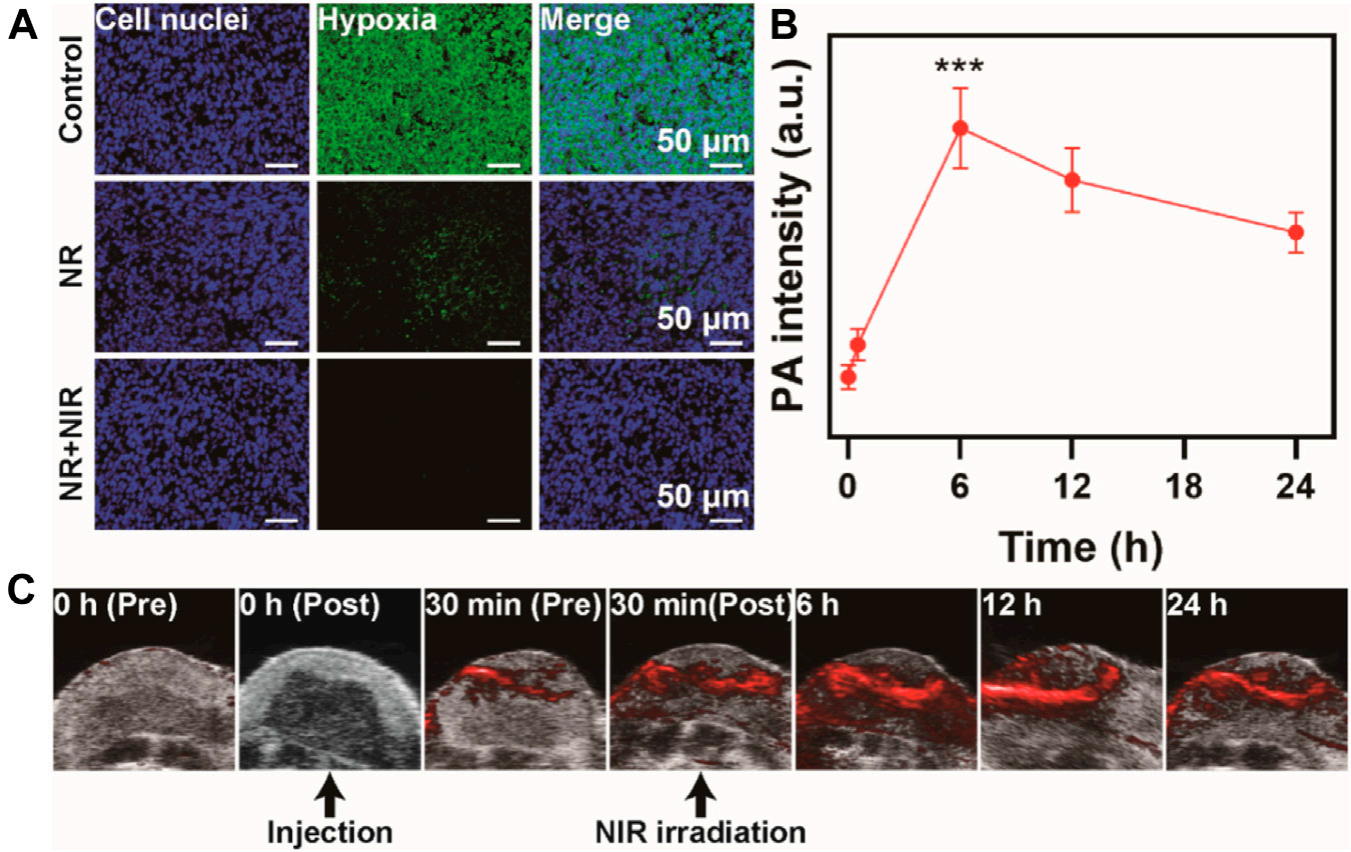
Hypoxia alleviation *in vivo*. **(A)** Immunofluorescence staining of tumor slices of mice. **(B)** PA signal intensity and **(C)** PA images at various time points. The data are represented as mean ± SD, ***p < 0.001.

Based on the remarkable catalytic ability and photothermal conversion ability of NR, we investigated the hypoxia-alleviating ability *in vivo*. Mice were divided into three groups and subjected to various treatments. For example, the mice in the NR + NIR group were intravenously injected with NR solution (50 μL, 100 μg/ml). Then an 808 nm laser was used to irradiate the tumor region 30 min after the injection. Next, the mice were sacrificed, and the tumor was sectioned for HIF-1α staining. After the injection of NR, the hypoxia was alleviated, which was due to the catalytic ability of NR to transform H_2_O_2_ to O_2_ (Figure 5A). Moreover, the mice subjected to the NR-mediated photothermal therapy showed the most obvious hypoxia alleviation among the three groups, which made it possible to fix the DNA damage induced by radiotherapy. PA imaging was then used to monitor the oxygenation status in the tumor region for 24 h. The intensity of the PA signal represented the oxygen level at 0 h; the mice received injection via tail vein. Then at 30 min post-injection, an 808 nm laser irradiation at a density of 2 W/cm^2^ was conducted for 1 min. The observation last from prior to the injection to 24 h post the injection. Next, 30 min post the injection of NR, the oxygen content rose, while after the irradiation, the oxygen level ascended significantly, the signal intensity of which reached 5.2 times as that of tumor irradiated immediately (Figures 5B,C **)**. Moreover, the intensity sustained at a relatively high level from that time until 24 h post-injection, indicating a sustained improvement of oxygenation in tumors.

**FIGURE 6:**
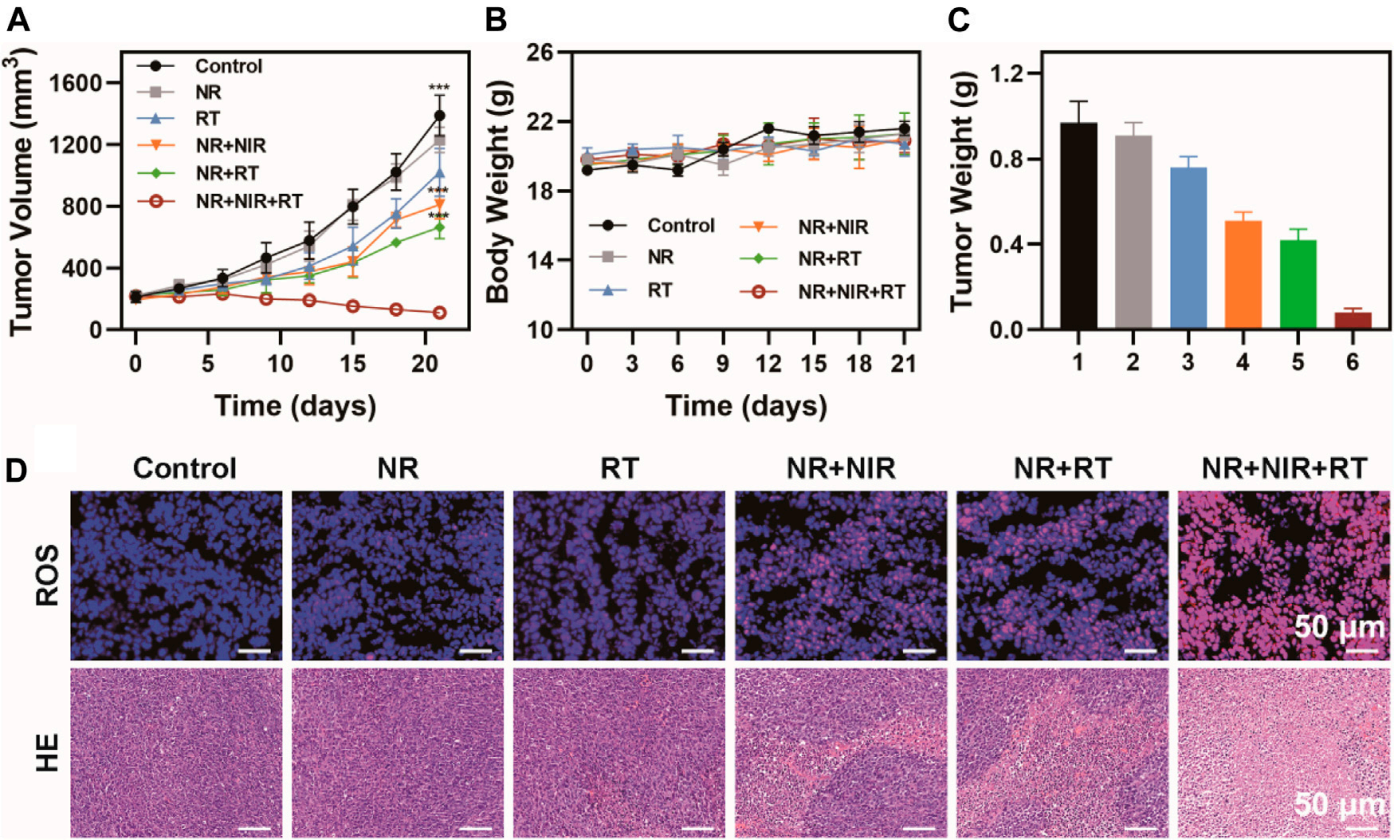
Antitumor efficiency *in vivo*. **(A)** Tumor volume, **(B)** body weight, and **(C)** tumor weight of mice in six groups (group 1: Control; group 2: NR; group 3: RT; group 4: NR + NIR; group 5: NR + RT; group 6: NR + NIR + RT); **(D)** Immunofluorescence staining of ROS and H&E staining of tumor slices of mice. The data are represented as mean ± SD, ***p < 0.001.

Based on these results, we further evaluated the antitumor efficiency on thermoradiotherapy mediated by NR. An MBT-2 subcutaneous tumor model on mice was utilized to verify the treatment groups. No apparent distinction was found in the body weight among all six groups, suggesting low toxicity of all the treatments (Figures 6A,B). However, the tumor grew rapidly in the control and NR group, while tumor growth was moderately restricted when treated with RT alone. NR-mediated photothermal therapy or NR-mediated radiotherapy exhibited relatively obvious tumor inhibition. However, among all the groups, thermoradiotherapy induced by NR exhibited the most effective tumor ablation ability, attaining a tumor growth inhibition value of 90.9% without any recurrence. Then the mice were sacrificed, and tumors were collected and weighed (Figure 6C **)**, which was consistent with the results of tumor volume. Furthermore, since ROS induced cell apoptosis, the immunofluorescence staining method was utilized to detect the ROS level of tumors in each group using a DCFH-DA probe. The group where the mice were subjected to NR-mediated thermoradiotherapy exhibited the strongest red fluorescence signal as expected, which indicated the highest level of ROS production among all the groups (Figure 6D **)**. Moreover, hematoxylin and eosin (H&E) staining was used to assess the status of cell apoptosis in all groups. After the treatment of NR + NIR or NR + RT, cell apoptosis in tumor tissues was of a relatively low level. However, by striking contrast, distinct cell apoptosis was observed in the NR + NIR + RT group. In addition, the hematological index of mice who received an injection of PBS or NR was assessed, which suggested remarkable biosafety of NR (Supplementary Figure S7).

## Conclusion

Here a PdTe NR was developed for hypoxia-alleviating thermoradiotherapy. The NR possessed nanoenzyme-like properties, and could effectively catalyze overexpressed intracellular H_2_O_2_ to O_2_. Also, the oxygenation status stayed at a high level from 6 to 24 h post-injection. Moreover, NR acted as an agent for photothermal therapy, which accelerated blood flow to boost sufficient oxygen to facilitate the DNA damage fixing induced by ionizing radiation. Since both Pd and Te are high Z elements, which can enhance the photoelectric effect to improve the energy deposition on tumor regions. The experiments results showed that this novel nanoplatform NR-mediated thermoradiotherapy could greatly inhibit the growth of MBT-2 tumors on a subcutaneous tumor model without obvious toxicity.

## Data Availability

The original contributions presented in the study are included in the article/Supplementary Material, further inquiries can be directed to the corresponding authors.
