# Supplementary material for: Hypoxia Alleviating PdTe Nanoenzymes for Thermoradiotherapy
Source: Front Bioeng Biotechnol. 2022 Mar 11;9:815185. doi: 10.3389/fbioe.2021.815185 (PMC8962630; doi:10.3389/fbioe.2021.815185)
Supplement: Supplementary file 1 [file DataSheet1.PDF]

Supporting information for

Hypoxia-alleviating PdTe nanoenzymes for thermoradiotherapy

Yang Li[1], Xinquan Gu[1]*, Fan Yu[2]*

[1] Department of Urology，China-Japan Union Hospital of Jilin University，18F，Section 7，No.126，Xiantai Street，Changchun，130033，China

[2] Department of Gartroenterology and Hepatology，China-Japan Union Hospital of Jilin University，9F，Section 6，No.126，Xiantai Street，Changchun，130033，China.

E-mail of Corresponding Author:

Xinquan Gu: guxq@jlu.edu.cn

Fan Yu: yufan2017@jlu.edu.cn

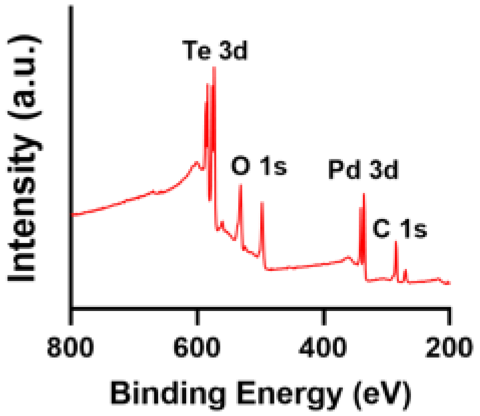

Figure S1. XPS survey of NR.

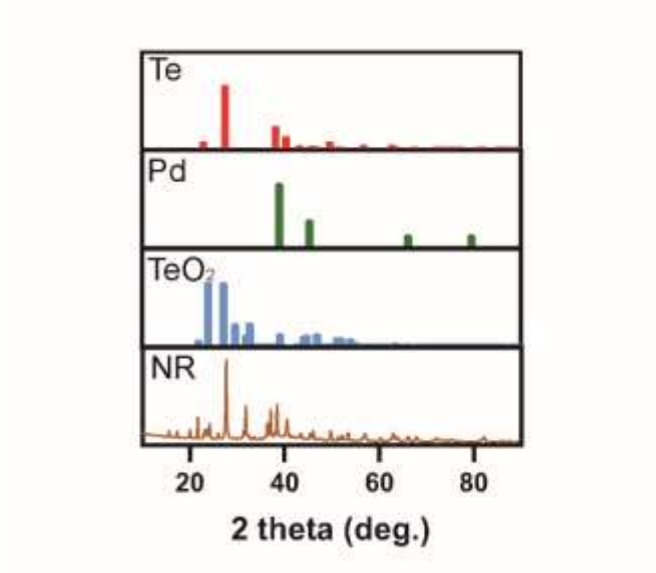

Figure S2. XRD powder patterns Te, Pd, TeO_2 and PdTe NR.

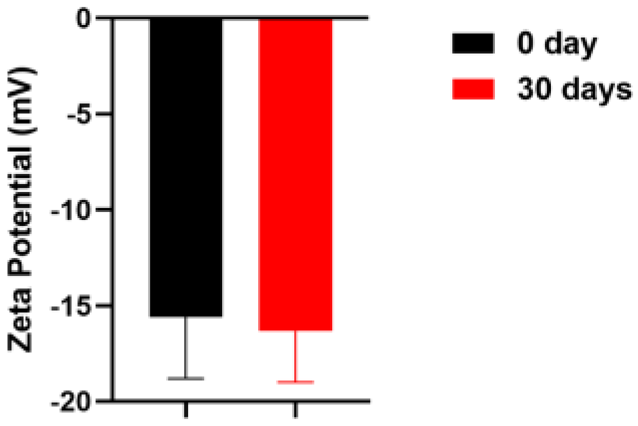

Figure S3. Zeta potential of NR at 0 day and 30 days post injection.

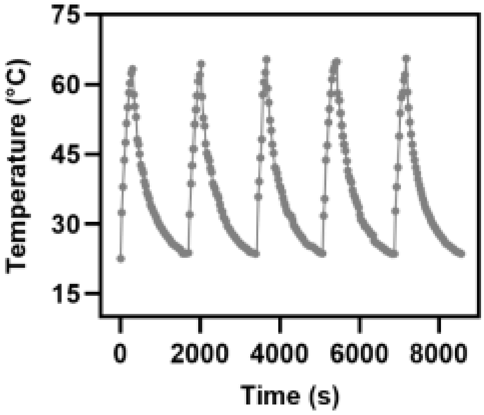

Figure S4. Heating and cooling curves of NR solution (100 μg/mL) at 808 nm laser irradiation at 1 W/cm^2.

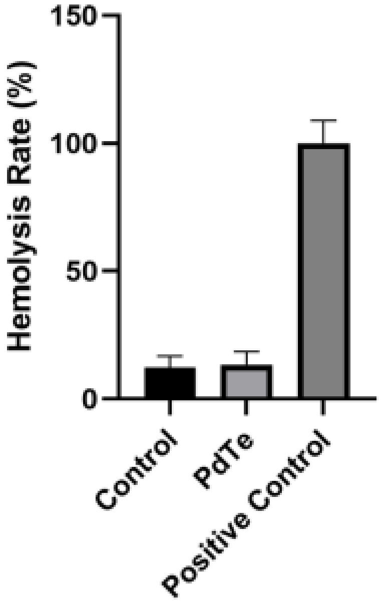

Figure S5. Hemolysis rate of PdTe.

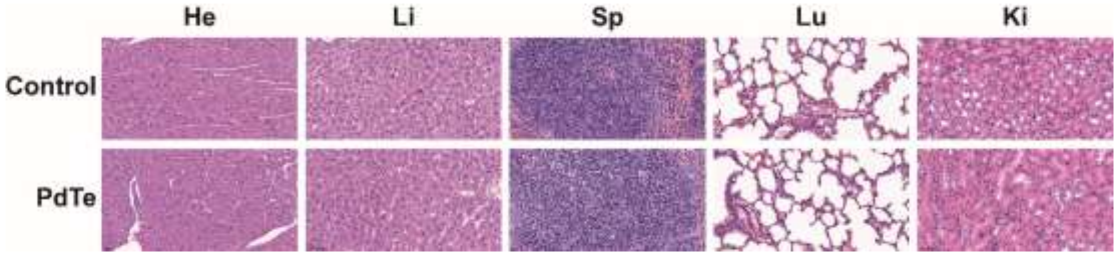

Figure S6. HE staining of main organs 30 days post injection.

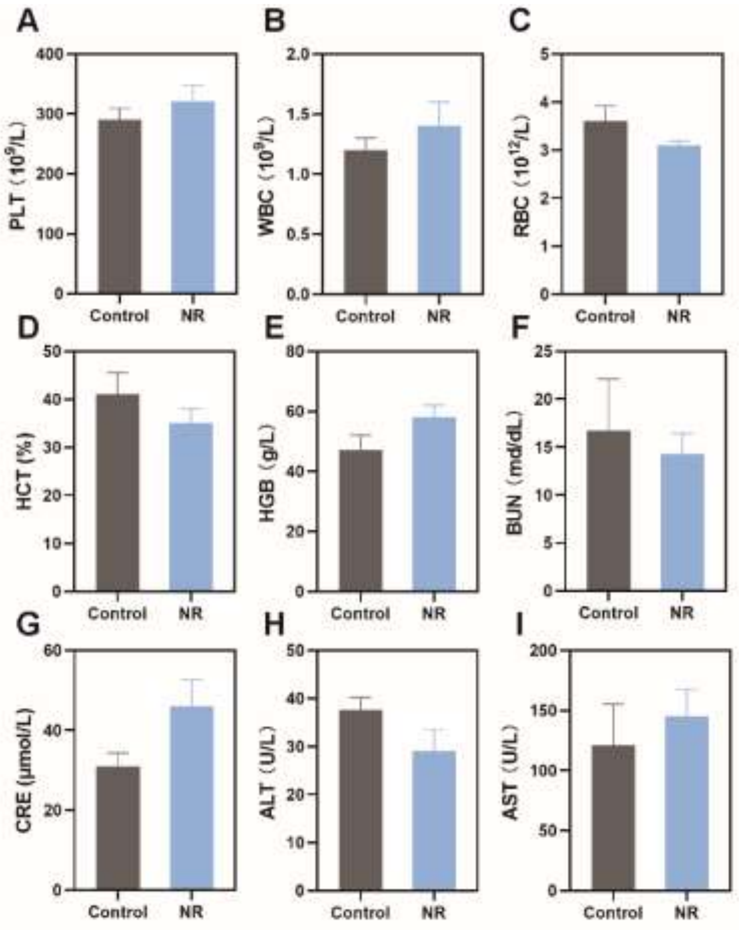

Figure S7. Hematological index of mice after 30 days post intravenous administration with PBS or NR (50 μL, 100 μg/mL) including (A) Platelet count (PLT), (B) white blood cell count (WBC), (C) red blood cell count (RBC), (D) hematocrit (HCT), (E) hemoglobin count (HGB), (F) blood urea nitrogen (BUN), (G) creatinine (CR), (H) alanine aminotransferase (ALT) and (I) aspartate aminotransferase (AST).

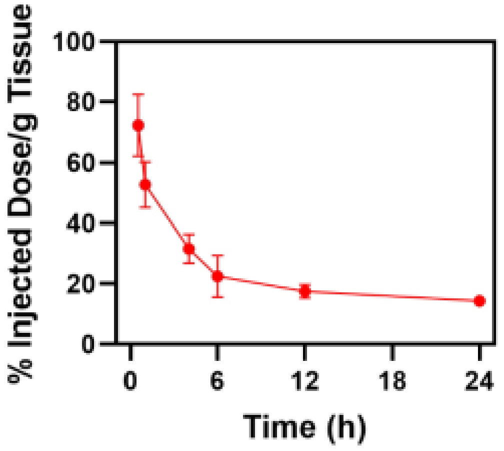

Figure S8. Blood circulation of NR in mice.

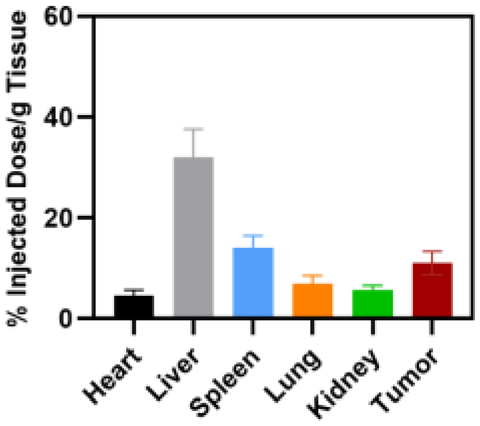

Figure S9. Biodistribution of NR in mice at 24 h post injection.

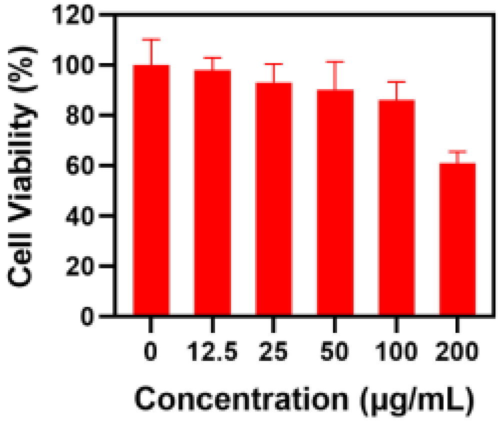

Figure S10. Cell viability after incubation with NR at various concentrations.
